# Supplementary figures and images for: Phosphorylation controls RNA binding and transcription by the influenza virus polymerase
Source: PLoS Pathog. 2020 Sep 3;16(9):e1008841. doi: 10.1371/journal.ppat.1008841 (PMC7494117; doi:10.1371/journal.ppat.1008841)

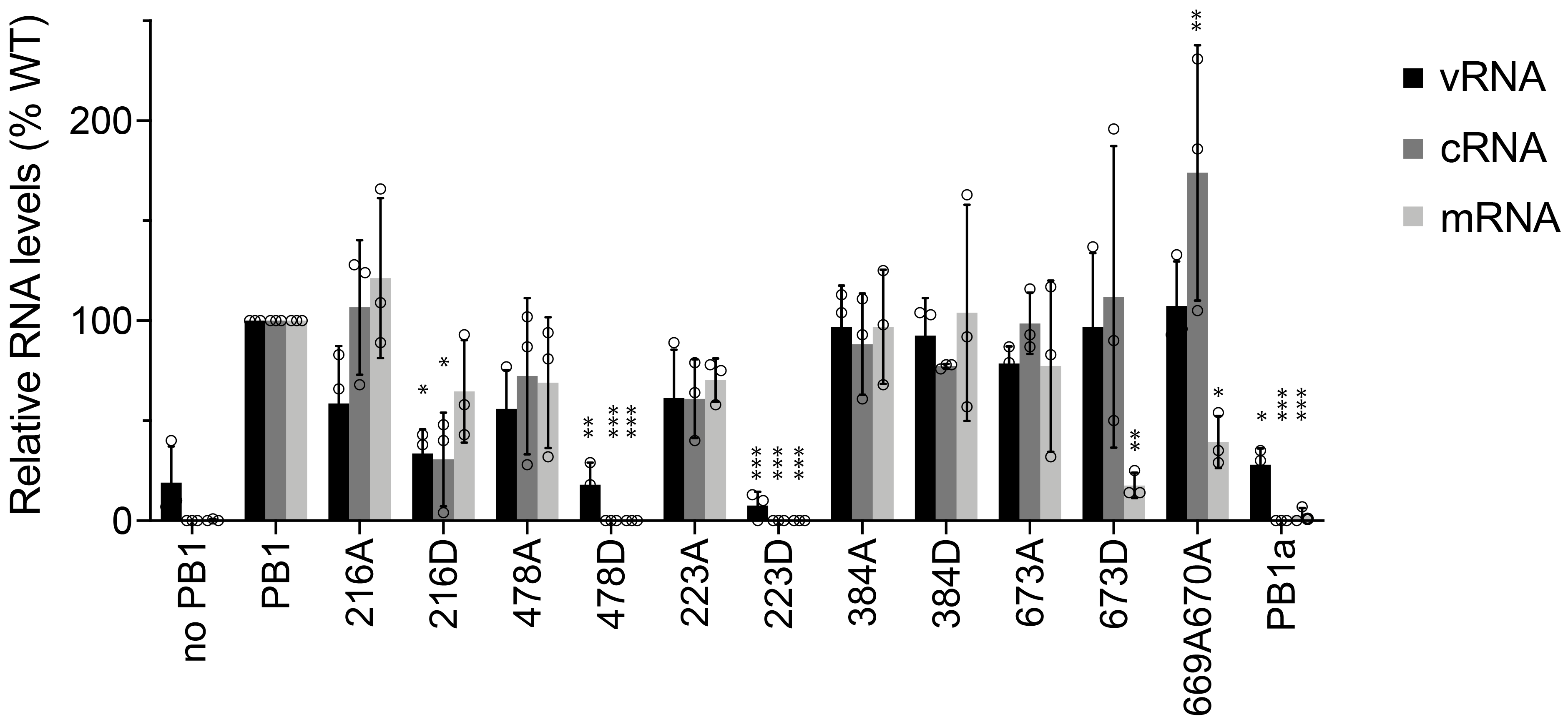

Supplement: S1 Fig — Three independent primer extension assays were quantified with each RNA species normalized to WT within an experiment. Data are presented at mean ± sd. * < 0.05, ** < 0.01, *** < 0.001 = P for one-way ANOVA with Dunnett’s post hoc compared to WT. (TIF) [file ppat.1008841.s001.tif]

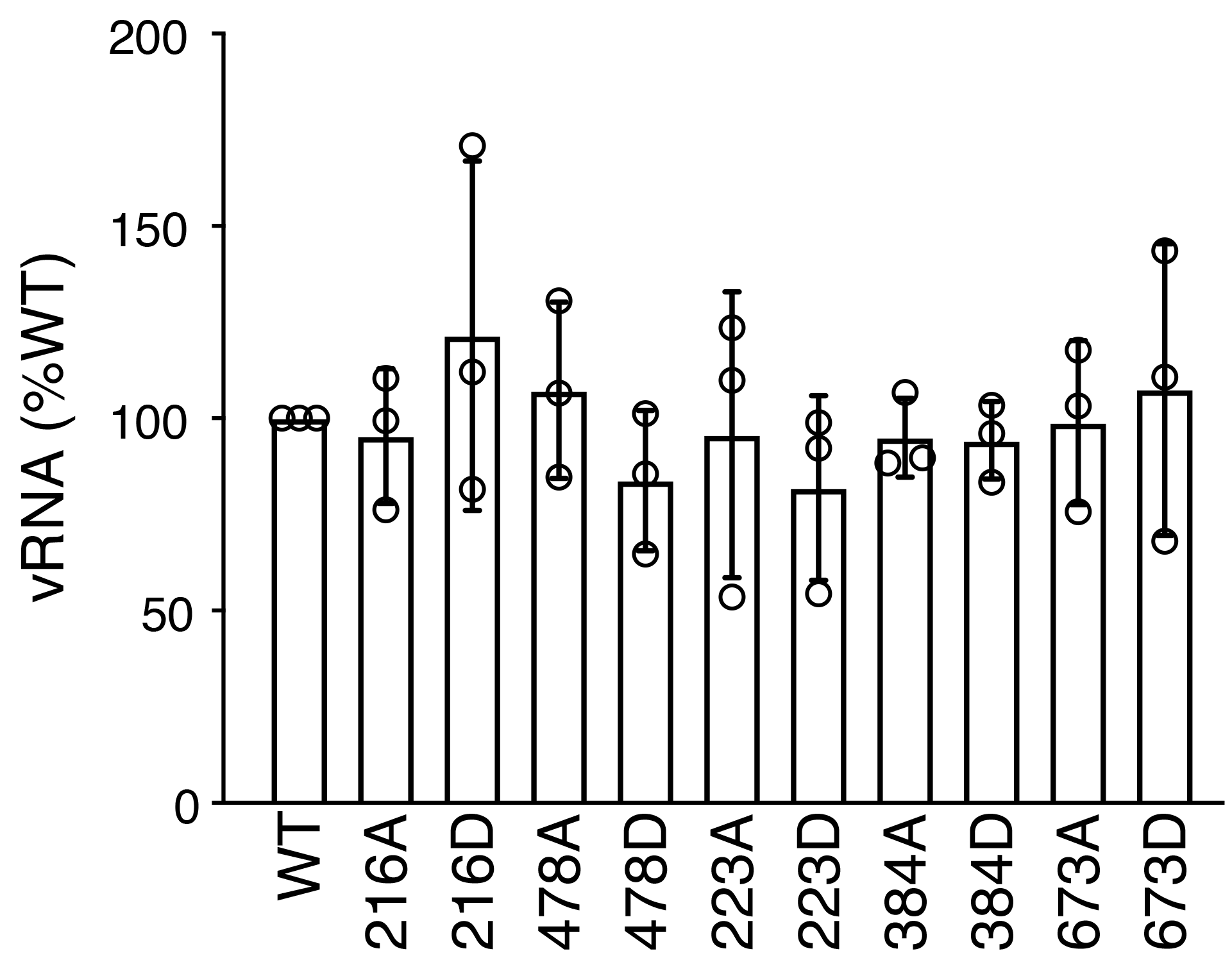

Supplement: S2 Fig — vRNA levels were quantified from three independent stabilization assays and normalized to WT. Data are presented at mean ± sd. There was no significant difference when analyzed by a one-way ANOVA. (TIF) [file ppat.1008841.s002.tif]

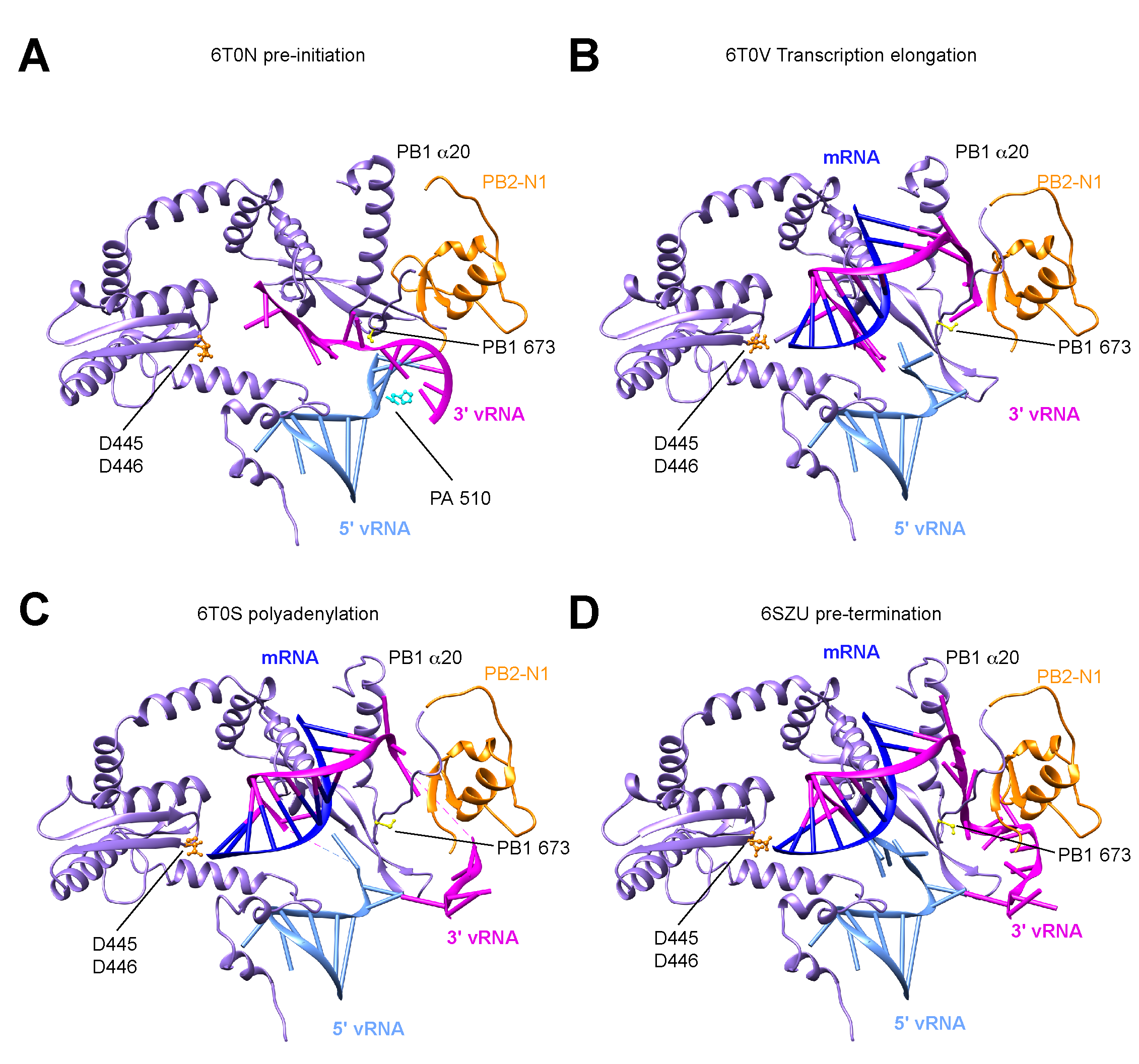

Supplement: S3 Fig — Structures of the polymerase at different stages of the catalytic cycle. Portions of PB1 are shown in purple with the motif C residues D445/D446 in the catalytic site in orange. 5’ vRNA is light blue, 3’ vRNA is magenta, and transcription product is dark blue. The PB2-N1 domain is modeled in orange. A. PB1 S673 (yellow) and PA H510 (cyan) flank the incoming 3’ vRNA template in a pre-initiation state (PDB 6T0N). B-D. Residues upstream of the PB1 α20 helix remodel to create the template exit channel used during transcription elongation (PDB: 6T0V). PB1 S673 is repositioned at the floor of the exit channel and remains part of the exit channel during later stages of transcription including polyadenylation (PDB: 6T0S) and pre-termination (PDB: 6SZU). Number and residue assignments are based on WSN sequences. (TIF) [file ppat.1008841.s003.tif]
