# Supplementary material for: Phosphorylation controls RNA binding and transcription by the influenza virus polymerase
Source: PLoS Pathog. 2020 Sep 3;16(9):e1008841. doi: 10.1371/journal.ppat.1008841 (PMC7494117; doi:10.1371/journal.ppat.1008841)
Supplement: Attachment — Submitted filename: Mehle_Response to reviewers.pdf [file ppat.1008841.s007.pdf]

Reviewer #1

This manuscript is an extension of the authors' 2017 study in eLIFE, which demonstrated that phosphorylation of the influenza viral RNA polymerase modulates viral genome replication by controlling the assembly of replication complexes. In this study, the authors used mass spectrometry to identify distinct PB1 phosphorylation sites that modulate polymerase activity and polymerase RNA binding. Analyses of numerous phospho-mimetic or phospho-ablative mutations in PB1 identified residues in PB1 that are involved in replication and in transcription of the viral genome. While this study is expertly executed, it is only incremental to what the authors published in in their 2017 eLIFE paper.

We thank the reviewer for their positive assessment of our data and conclusions. As the reviewer notes, this manuscript was submitted as a Research Advance directly tied to our prior paper. Our 2017 study demonstrated that PKC δ-mediated phosphorylation of NP blocks NP oligomerization and that PKC δ is required for efficient RNA synthesis requiring *de novo* RNP assembly. The current study builds on the importance of phospho-regulation, but identifies new mechanisms. Here we show that the viral polymerase is phosphorylated and these modifications directly regulate polymerase activity. Combined, our 2017 eLife paper and this submission reveal two modes of phospho-regulation:

1) control of NP oligomerization which affects RNP assembly and indirectly controls genome replication
2) direct regulation of polymerase output by phosphorylation of the PB1 subunit

Thus, while the papers share the theme of phospho-regulation, their conclusions and underlying mechanisms are distinct. Moreover, this is the first demonstration of a discrete regulatory effect of phosphorylation on influenza polymerase activity.

Reviewer #1 (Minor Comments):

1. Figure 1: Do studies in cells that express a constitutively active PKCd recapitulate what is happening in normal cells?

We had previously shown that PKCδ regulates influenza virus RNP function. Those initial experiments also suggested that the viral polymerase might be phosphorylated. Using those prior hints as motivation, Fig 1 in the current study confirms those initial observations and shows that active PKCδ modulates polymerase activity. We agree with the reviewer that this is may not fully represent “normal cells.” However, we have shown that PKCδ is activated during infection (Mondal et al 2017), supporting this approach. The main objective was to establish that the polymerase can be phosphorylated and the these events alter its activity. With these proof-of-principle data in hand, we then pursued the more challenging experiments in Fig 2 to identify phospho-sites in multiple conditions, including those that occur during infection of “normal cells.”

2. Some identified phosphorylation sites were identical in experiments carried out in plasmid-transfected and infected cells. Why did'nt the authors focus on studying roles for PB1 phosphorylation sites that were only observed during infection?

We detected 13 different phosphorylation sites in 5 distinct experiments. We were most interested in sites that may directly affect polymerase activity, and not other functions of the polymerase like RNP export and trafficking. Thus, we focused on high confidence sites identified in polymerase activity assays (when the primary function is polymerization and we had functional data showing PKCδ expression altered function), most of which were also found in infection (to ensure biological relevance). We now clarify this reasoning in the text on lines 138-145.

3. Why does mutant S673A produce more NP mRNA than wt (Fig. 3A), but less in the primer-extension analyses shown in Fig. 4A. Comparison of these two figures suggests that RT-PCR (Fig. 3A) and primer-extension analyses (Fig. 4A) produce different results. Which ones are correct?

This is an astute observation, and the differences in RNA abundances detected in Figure 3A and 4A most likely stem from differences in experimental approach. Figure 3A reports polymerase activity in a single-cycle infection, whereas Figure 4A reports RNA levels in a polymerase activity assay in transfected cells. Both figures provide important, but slightly different information. Figure 3A reveals RNA levels during an infection. But, it is limited as not all PB1 mutants were capable of creating infectious virus. Figure 4A avoids this limitation, but in this minimalist system other regulatory events may be missing. We are not the first to notice these assay-specific difference (see Robb et al 2009). This is why we performed both approaches and integrated data across experiments to make our larger conclusions. We note these differences in the text on lines 183-190.

4. Fig. 5B.: The abundances of vRNAs are difficult to judge, because the input samples contain different abundances of vRNAs. This needs to be carefully quantitated.

This experiment tests whether a polymerase can still bind viral RNA. It very clearly shows that PB1 T223D is unable to precipitate viral RNAs, even if there are minor variations in input vRNA. We are not trying to make strict quantitative comparison. Nonetheless, we now provide a ratio of the quantified radiolabel from the RIP and input samples under the gels in Fig 5B.

Reviewer #2

Dawson et al. analyze phosphorylation sites in the PB1 protein of influenza virus polymerase. The authors use mass spectrometry to identify phosphorylated PB1 residues and analyse their effects on polymerase assembly, RNA synthesis and virus

growth. They demonstrate that the phosphorylation status of some sites appears to affect viral growth and/or RNP activity.

Overall, the data presented in the paper supports the authors' conclusions. However, we think, that the discussion of the differences observed between phosphorylation sites identified during viral infections and RNP reconstitution is lacking.

Overall, this is solid work that will be of relevance to readers interested in the details of influenza polymerase biochemistry. However, we question whether the broader biological relevance will appeal to the broader audience of eLife.

We thank the reviewers for their complimentary assessment of our "solid work that will be of relevance to readers interested in the details of influenza polymerase biochemistry."

Major comments:

Authors identify several residues that are phosphorylated in the context of infection but not transfection (T228, Y253, T570, S712, and S720). The authors do not follow up on these residues and it is not clear if they attempted to rescue viruses with phospho mimetic or ablative mutants at these sites. Could authors comment on this choice and explain why they choose to emphasise more the phosphorylated residues that are identified in RNP reconstitutions (even the ones that are not present in the context of viral infection) given the "simplified nature" of transfection assays which may not fully reflect events in a biological context.

The reviewer indicates that we did not sufficiently discuss differences in phospho-patterns between infection and polymerase activity assays. This is similar to a comment from Reviewer 1. As we noted above:

> We detected 13 different phosphorylation sites in 5 distinct experiments. We were most interested in sites that may directly affect polymerase activity, and not other functions of the polymerase like RNP export and trafficking. Thus, we focused on high confidence sites identified in polymerase activity assays (when the primary function is polymerization and we had functional data showing PKCδ expression altered function), most of which were also found in infection (to ensure biological relevance). We now clarify this reasoning in the text on lines 138-145.

Given this focused scope of the current submission, we did not attempt to rescue viruses harboring phospho-mimetic/ablative mutations at the remaining site in PB1.

The authors place a lot of emphasis on the fact that phosphorylation at PB1 S673

biases plus-sense RNA synthesis from transcription to replication, but at the same time their phospho-ablative mutants in the same residue position show very different phenotypes in the context of infection and RNP reconstitution. How do they know that their S673D mutation is reflective of the effects they'd expect to observe in the context of other viral genes? The authors only provide a sentence on line 186-188 saying the assays gave different results for some mutants.

Our primer-extension data show that a phospho-mimetic at PB1 S673 suppresses transcription. We were unable to rescue virus encoding PB1 S673D, despite multiple attempts and our prior ability to rescue very attenuated mutants. Thus, RNP reconstitution assays (Fig 4) and pre-expression of mutant prior to infection (Fig 5) are close approximations given such strong defects. Similar approaches are routinely used in the field (see examples in Hutchinson…Fodor 2012 and later Turrell…Fodor 2015 where they use these techniques to influenza NP phospho-mutants that cannot be rescued as infectious virus). The fact that PB1 S673D exhibits strong changes in polymerase output is consistent with its inability to support virus production.

The reviewer also correctly notes that quantification of mRNA in single-cycle infection versus RNP reconstitutions are not always concordant, with PB1 S673A showing increased mRNA during infection but WT levels in RNP reconstitutions. We are not the first to notice these assay-specific difference (see Robb et al. 2009). This is why we performed both approaches and integrated data across experiments to make our larger conclusions. We now expand our discussion of their limitations on line 183-190.

In Figure 3, can the authors comment on why S478A is attenuated for growth despite no effect on polymerase activity?

This is an interesting question. Both the gene expression assays (Fig 3) and polymerase activity assays (Fig 4) measure the ability of mutant polymerases to produce canonical viral RNA products (vRNA, cRNA, and mRNA). This only reflects its function as a polymerase, and not its other functions during the life cycle, which includes RNP export, trafficking and packaging. In addition, mutant polymerases may lead to the production of other RNA products not detectable in these assay. Some mutant polymerases are known to create immunogenic mini-viral RNAs or defective interfering RNAs that may explain the unusual growth curves (Fodor, et al. 2003; te Velthuis, et al. 2018). Given the speculative nature of these possibilities, we present this as a possible explanation in the discussion on line 300-306.

In the right panel of Figure 3B, there are no data shown for wildtype.

We apologize for the misunderstanding. All mutants in Fig 3B were analyzed in the same experiment. However, graphing them all together made it difficult to interpret, so we segregated the phospho-mimetics. We clarified the figure legend to indicate that the

WT curve applies to both graphs.

Reviewer #2 (Minor Comments):

Figure 1 needs size markers.

We added size markers as requested.

Can the authors explain why in the context of infection whole cell lysate and IP-NP samples identify completely different phosphorylation sites? These samples are probably not comparable given that WCE is done for a specific time point and IP-NP is done on pooled time course, but would you expect a pooled WCE sample to give results more like pooled IP-NP? If so, why choose to do WCE for single time points? It is possible that the phosphorylation sites depend on the differences of sample preparation method.

We agree with the reviewer that the experimental design will affect the phospho-sites that can be detected. It is further complicated by well known run-to-run variability in mass spectrometry analyses. This is why we used multiple approaches and replicates. Differences in identification between whole cell extracts (WCE) and IP-NP samples are possibly due to a number of factors. A major driving factor is the completely different approaches. WCE is a very complex sample where viral proteins are minor components, whereas the NP IP sample contains concentrated and partially purified viral proteins. The enrichment in the NP IP sample likely explains the better depth of coverage. In addition, the NP IP samples only contain NP-associated polymerases, whereas whole cell lysate encompasses both NP-free and NP-associated polymerase. For these reasons, we would not expect IP and WCE samples derived from the same time point to necessarily be comparable.

Although the authors spend the first part of the manuscript emphasizing phosphorylation sites in PB2 (Figure 1, the analysis is then switched to PB1. Are data for all the the polymerase from the mass spec is available).

The reviewer is correct that this analysis will also detect phosphorylation of the other polymerase subunits. We chose to focus on the catalytic subunit PB1 for this manuscript. Analysis of phospho-sites in PB2 and PA is the focus of a future manuscript. That said, we are happy to share the mass spectra upon request and make this explicit in the methods on line 443.

From the methods It's not clear how the cells expressing constitutively active form of PKCδ that are used in Figure 1 were generated. Where the cells just transfected with PKCδ, like it is done for mass spec, either way clarify).

We clarified the methods to indicate that cells were transiently transfected to express constitutively active PKC δ. See new methods subsection on lines 343-348.

Given that there are not that many phosphorylated sites and identification of such sites is the major goal of the paper, Figure 2 should include a summary table that makes clear which sites are phosphorylated in the context of infection, transfection or both (something similar or even the same as now in supplementary table).

These data were detailed in the original Table S1. We have now updated Figure 2B to indicate which sites were found during infection.

In the methods section, please provide a description of how the amino acid conservation in figure 2 was calculated.
We updated the methods section to describe our amino acid conservation analysis, beginning on line 475.

On line 107 it's vNP77 short template in methods line 381 it's vNP76 template, please correct for which it is.
We corrected this to accurately reflect the short template as vNP77. Our version contains one additional nucleotide relative to that first described by Turrell, et al.

Can the authors comment on the strange way that the phospho-ablative mutants affect viral growth, with titers reaching the same peak at 48 hours but then decaying more quickly than wildtype. Does this suggest reduced stability of the mutant virions?

We too noticed this unusual growth curve. We obtained similar curves across multiple biological replicates. As we alluded to above, it is possible that polymerase mutations affect other activities that we did not directly investigate. The growth curves could potentially arise from the fact that some mutant polymerases are known to create immunogenic mini-viral RNAs or defective interfering RNAs that alter replication (Fodor, et al. 2003; te Velthuis, et al. 2018). Given the speculative nature of these possibilities, we present this as a possible explanation in the discussion on line 310-316.

The authors state in the text that viruses with phospho-mimetic mutations at sites 673, 223, and 478 could not be rescued, but it would help clarity to mention this in the main text.

The original submission states in the main text that “phospho-mimetic mutants at PB1 T223, S478 and S673 failed to yield virus despite multiple attempts.” See lines 154-155.

The wording in line 162-164 is confusing. The authors say "These viruses" and "all viruses" in a way that makes it sound like they are referring to all the mutants, but then in line 168 clarify that this doesn't include all the viruses since some didn't grow at all.

Thank you for pointing this out. We clarified the text indicating that all rescued viruses were assessed for multicycle replication.
