# Supplementary material for: Phosphorylation controls RNA binding and transcription by the influenza virus polymerase
Source: PLoS Pathog. 2020 Sep 3;16(9):e1008841. doi: 10.1371/journal.ppat.1008841 (PMC7494117; doi:10.1371/journal.ppat.1008841)
Supplement: Attachment — Submitted filename: PLOS P Rebuttal final.docx [file ppat.1008841.s008.docx]

**Part I - Summary**
Please use this section to discuss strengths/weaknesses of study, novelty/significance, general execution and scholarship.

We thank reviewer #1 and #2 for considering this revision and their helpful comments. We especially appreciate the insight of reviewer #3. This has allowed us to incorporate new data that has been published since our original submission, making the submission more timely and relevant.

Reviewer #1: The authors have adequately addressed previously raised concerns.

Reviewer #2: In this paper Dawson et al. identify multiple phosphorylation sites in the PB1 subunit of influenza A virus polymerase using mass spectrometry. Using RNP reconstitution and primer extension assays the authors demonstrate mechanistic effects of PB1 phosphorylation on the ability of viral polymerase to transcribe and replicate viral RNA. Finally, using viral reverse genetics, the authors also show that some of the discovered phosphorylation sites are required for virus replication in tissue culture.

Overall, the presented research supports the authors’ conclusion and presents findings about the modulation of viral genome replication by viral polymerase phosphorylation that will be of interest to the influenza research community.

Reviewer #3:  The authors use mass spectrometry to identify phosphorylation sites of influenza virus polymerase, either transfected together with active PKCdelta or in infected cells. Three out of 13 sites in PB1 are common to both kinds of experiment. They focus on five sites and by analysing phospho-mimetic and phospho-ablative mutants, in recombinant viruses or transfections, show that the phosphorylation status modulates polymerase activity in diverse ways. Such information is new and beneficial, as a starting point, for improving understanding of polymerase regulation during viral infection. However, in view of the vast amount of structural information now available on influenza polymerase in different functional states, especially during transcription, the discussion of the mechanism of these phosphorylation effects is disappointing and should be further developed. Structural studies show that the polymerase is incredibly flexible, locally and globally, especially in the apo-state, and has multiple interchanging functional conformations. This undoubtedly affects accessibility of certain residues to kinases and phosphatases. Furthermore, many residues have multiple roles. For example, PB1 S673 helps stabilise a particular conformation of the 3’ end in the promoter, but during elongation it is in the outgoing template channel. Similarly, PB1 T570, which the authors unfortunately do not study in detail, is in the newly described secondary 3’ end binding site and its phosphorylation could inhibit RNA binding there. Residues S712 and S720 are both in the interface between the PB1 core and the PA endonuclease domain, but this is not mentioned.

We thank the reviewer for his/her constructive feedback and the opportunity to expand our analysis. Several new structures have been released since this paper was originally submitted in February. Our current findings have direct bearing on these new structures, highlighting the importance of this submission. We respond in detail below as to how we have incorporated these new structures into our analysis, including new panels in Fig 2, highlights of relevant structural changes in Sup Figure 3, changing patterns of accessibility of phospho-sites as the polymerase cycles through various stages in supplemental table 2, and assigned functions for all phospho-site in the Summary Data tab of Supplemental table 1, including those not studied extensively in this submission

**Part II – Major Issues: Key Experiments Required for Acceptance**
Please use this section to detail the key new experiments or modifications of existing experiments that should be absolutely required to validate study conclusions.

Generally, there should be no more than 3 such required experiments or major modifications for a "Major Revision" recommendation. If more than 3 experiments are necessary to validate the study conclusions, then you are encouraged to recommend "Reject".


Reviewer #1: (No Response)

Reviewer #2: No major comments. Authors have appropriately addressed the previous major from the reviewers and updated their manuscript accordingly.

Reviewer #3: 1. The authors make poor use of available structural information to help the reader think about possible effects of the observed phosphorylation sites. Rather than just categorising as catalytic or template entry, they should provide a table with a short description of the structural position of the residue and its possible functional role

We agree with the reviewer; multiple new structures have been released since this paper was originally submitted and we are happy for the opportunity to include these in our discussion. We have updated the Summary Data tab in Supplemental Table 1 with possible effects of phosphorylation at the indicated residues, including those not studied in depth in this submission.

2. Figure 2B should be improved to show all identified sites in the global view and the detailed part on top of the global view should be removed. There could be two enlarged view, one of the promoter bound form, one of the transcription elongation form.

We have updated Figure 2 to show the general location of each phosphosite in the context of vRNA-bound polymerase and polymerase during transcription elongation. Detailed renderings of the catalytic core are also provided. New Supplemental Figure 3 provided even more focus on residue S673 and the multiple positions it assumes as the polymerase transitions through different stages.

3. The authors should comment on the accessibility of the individual residues early on (and not only in the discussion), to give the reader an impression how reversible individual modifications might be. The authors should not just say ‘residues are not immediately on the surface’ but calculate solvent accessibility in different polymerase states (compare for example the transcriptionally ready 4WSB and the cRNA bound 6KUJ, or the recent polymerase dimers e.g. 6QX8). The authors might also emphasise that some of the sites (especially those around RNA binding regions) are probably more accessible when the polymerase is RNA free.

We have included additional analysis of surface accessibility of each residue in the context of multiple polymerase complexes (Supplemental Table 2). The results section now includes data on possible accessibility of these residues (Lines 157-164). We note that residues corresponding to WSN PB1 T223, T385, S478 and T570 were inaccessible in all conformations analyzed, PB1 S384 was accessible in all conformations, and S673 was buried in the vRNA-bound structure but may be accessible in the monomeric. The remaining sites displayed an intermediate degree of accessibility or could not be fully assessed as regions of PB1, PA and PB2 that would affect accessibility were not resolved in some structures. We suggest that highly-accessible residues may be dynamically modified, whereas the modification status of residues that are buried in a particular conformational state may be static.

4. The authors find T223D eliminates vRNA binding and cRNA stabilisation, yet, according to structures, it is not in close contact with viral RNA. On the other hand S673 directly binds the promoter 3’ end and thus S673D would be more expected to effect vRNA binding. The authors should perform the same vRNA and cRNA binding studies on the S673D mutant and try to explain this counter-intuitive phenotypes of these two mutations.

We now highlight the surprising result that S673D retains RNA binding despite being in closer proximity to RNA templates and provide an extensive discussion on possible reasons suggested by newly solved structures of the polymerase (paragraph beginning on line 306). In brief, S673 is poised to impact RNA binding in a pre-initiation state, or to affect the template exit channel that forms during transcription. If the later is correct, this would explain why we are competent to bind RNA and replicate genomes, but not transcribe. Both possibilities are discussed.

Our functional data in Figures 4A, C and 5A-B demonstrate that PB1 S673D produces replication products and stabilizes cRNA similar to WT. This outcome requires that PB1 S673D is competent to bind vRNA and cRNA. This is supported by prior work with the PA H510A mutant polymerase (Fodor 2002). PA H510 and PB1 S673 are on opposite sides of the 3’ vRNA in the pre-initiation structure. The PA H510A mutant exhibits the same phenotype as PB1 S673D where it can replicate, but not transcribe. We therefore do not feel the additional experiments requested here will advance our understanding of PB1 S673 function.

5. Lines 275-276. The authors suggest there is evidence that the 3’ end of the template is differently located dependent on whether it is undergoing transcription or replication initiation. Specifically, from its structurally observed position in the active site, the 3’ end appears to need to backtrack by one nucleotide to allow terminal initiation of vRNA to cRNA replication, although using the same reasoning (based on the pathway of the template going into the active site being always the same), would apparently not need to do so to allow internal initiation of cRNA to vRNA initiation. The observation that S673D specifically affects transcription but not either sense of replication suggests that, given its key structural role in stabilising the G9-C8 kink (which would seemingly be totally disrupted if S673 were phosphorylated) in the observed promoter structure, the template pathway may not be the same and indeed the whole promoter might be different during replication. This is a very significant observation that merits more careful discussion (and a figure showing the dual roles of S673 in promoter binding and in elongation). For instance, another nearby residue, intimately involved in binding the promoter, PA H510, upon mutation also specifically affects transcription (J Virol. 2002;76(18):8989‐9001. doi:10.1128/jvi.76.18.8989-9001.2002).

We agree with the reviewer and, as mentioned above, have now expanded our discussion of S673D and have provided an additional supplemental figure mapping 673 during various stages of the transcription cycle. We thank the reviewer for pointing out the PA H510A mutant. The potential roles of phosphorylation of S673 during transcription are discussed in the context of this previously characterized transcription-deficient PA H510A. We also speculate on the role of S673D in the newly described template exit channel used during transcription (Lines 306-335).

**Part III – Minor Issues: Editorial and Data Presentation Modifications**
Please use this section for editorial suggestions as well as relatively minor modifications of existing data that would enhance clarity.


Reviewer #1: (No Response)

Reviewer #2: No minor comments. Authors have appropriately addressed the previous minor from the reviewers and updated their manuscript accordingly.

Reviewer #3:

1. Remind the reader whether PKCdelta is active in the nucleus or cytoplasm or both.

We have added a brief comment noting that PKC delta is activated during infection, it localizes throughout the cell, and how virally induced apoptosis may extend its localization and activity (Lines 95-100)

2. Is it possible to quantify from the mass-spec analysis the fraction of polymerases that have a particular phosphorylation?

Although this information would be quite useful, it is not possible to know the fraction of polymerases that have a particular phosphorylation. We performed phospho-peptide enrichment to enhance our sensitivity. This removes the unphosphorylated peptides that would be needed to estimate the fraction that is phosphorylated.

3. Expand the phospho-residue identity search to bat influenza A and influenza B. Sites really important for modulation of intrinsic functions are likely to be fully conserved. For instance S384, whose A/D mutations are pretty much like wild-type, is already an aspartate in bat influenza polymerase.

The table present in Figure 2A is now expanded to included bat influenza A and influenza B and the data are discussed on lines 154-155.

4. On lines 268-284 the authors describe the effects of S673 phosphorylation. Indeed, the S673 phosphorylation may influence the behaviour of template RNA in this region. However speculation in lines 281 about charge neutralisation is not supported by the structure organisation of the region.

We have included further discussion of the template exit channel (lines 319-332). The basic nature of this channel was highlighted in our original submission based on older mutagenic studies showing K669/R670/R672 were important for transcription (Kerry 2008). We have now included the most recent structure that shows this region creates the basic floor of the exit channel during transcription (Wandzik et al 2020) and suggest the negative charge of phosphorylation may disrupt the positive charge in this region.

5. The authors speculate that some of the inaccessible sites might be modified already during polymerase translation and are static/irreversible (lane 307). The T223 and S478 are actually an example of inaccessible residues. More over the S478D is completely inactive. How do the authors explain what happens when such a polymerase is incorporated into a newly made RNP (lane 289) thus making it incapable of any further transcription and replication. Such a contradiction should be explained in comparison to positive effect of activation of RNPs in trans (lane 293).

We now note potential consequences S478 phosphorylation beginning on line 341:

S478 lies in the NTP channel of the polymerase and phosphorylation would likely interfere with NTP transit or positioning [46]. This polymerase is catalytically inactive, but like PB1a may still impact overall polymerase output by functioning in *trans* to stimulate cRNP activity [19]. Conversely, RNPs harboring PB1 phosphorylated at S478 would be non-productive; thus phosphorylation may be an antiviral host response. It remains to be determined whether phosphorylation of PB1 S478 exerts antiviral activity or contributes to *trans* polymerase function during infection.

6. Line 302-304 unsupported speculation whose relevance is dubious.

We have removed this statement.

7. Line 306. This is a very static view of the polymerase, whereas it is actually extremely flexible.

We have rephrased this line to convey our intended meaning that the modification, and not the polymerase itself, may be static (Lines 36-363).
